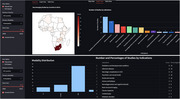# Mapping Neuroimaging Capacity in Africa

**DOI:** 10.1002/alz70861_108454

**Published:** 2025-12-23

**Authors:** Charity Umoren, Maruf Adewole, Ayomide Oladele, Olujide Oyeniran, Udunna Anazodo

**Affiliations:** ^1^ Medical Artificial Intelligence Laboratory, Crestview Radiology Ltd, Lagos Nigeria; ^2^ Medical Artificial Intelligence Laboratory, Lagos, Lagos Nigeria; ^3^ Montreal Neurological Institute, McGill University, Montreal, QC Canada; ^4^ Medical Artificial Intelligence (MAI) Laboratory, Crestview Radiology Limited, Lagos Nigeria

## Abstract

**Background:**

Globally, neuroimaging is essential in the diagnosis and treatment of brain disease, including dementia. Its use in assessing disease biomechanisms has gained increased popularity in neuroscience over the last two decades. However, the capacity for translational neuroimaging research in Africa is limited and has not been systematically characterised. This study aims to understand, quantify, and analyze the evolution and use of neuroimaging modalities for research in Africa. A scoping review was performed to identify the neuroimaging research challenges in the region, evaluate common imaging modalities and indications in the region, characterize the studied population, and outline opportunities to close capacity gaps.

**Method:**

A scoping review of human brain imaging studies in Africa was conducted following the Joanna Briggs Institute (JBI) Manual for Evidence Synthesis guideline and using a pre‐registered protocol [osf.io/k7qw6]. A Participants, Concept, and Context framework [osf.io/nuf2m] was used to identify relevant original studies from the past 20 years. Data describing the studies’ population demographics (age, sex, diagnostic status, ethnicity, etc.,), duration, geographical location, neuroimaging technology (modality, scanner details, contrast use, sequences, etc.,), authorship, and research output was extracted and is currently being analyzed. An interactive data visualization dashboard (**Mongoøse**) was developed to graphically summarize the data, map geographical distribution of studies, quantify imaging capacity, and reflect inter‐regional and cross‐continental collaborative research networks.

**Results:**

A prototype of the Mongoøse dashboard featuring multiple interactive elements and search filters, including choropleth maps of distribution of neuroimaging studies per country, tabular and graphical summaries is highlighted (Figure 1), to demonstrate the potential for assessing the region’s imaging research capacity. The full interactive dashboard is being developed based on the prototype with complete results of data analysis. The dashboard will offer a comprehensive overview of neuroimaging research capabilities in Africa: determine required capacity, measure the current capacity, identify capacity and representation gaps, and highlight opportunities for capacity building and international collaboration.

**Conclusion:**

This study will offer insights into alignment of Africa’s brain imaging resources with its neurological health challenges, including dementias. The findings will support global equity in neuroscience research and inform the development of sustainable neuroimaging infrastructure in resource‐constrained settings.